# Prognostic Value of Normal Stress Echocardiography in Obese Patients

**DOI:** 10.1155/2014/419724

**Published:** 2014-08-31

**Authors:** Michele Murphy, Siva Krothapalli, Jose Cuellar, Somsupha Kanjanauthai, Brian Heeke, Pallavi S. Gomadam, Avirup Guha, Vernon A. Barnes, Sheldon E. Litwin, Gyanendra K. Sharma

**Affiliations:** Division of Cardiology, Medical College of Georgia, Georgia Regents University, 1120 15th Street, BBR 6518, Augusta, GA 30912-3105, USA

## Abstract

*Background.* Chest pain is a common problem in obese patients. Because of the body habitus, the results of noninvasive evaluation for CAD may be limited in this group.* Methods.* We reviewed the records of 1446 consecutive patients who had undergone clinically indicated stress echocardiography (SE). We compared major adverse cardiac events (MACE; myocardial infarction, cardiac intervention, cardiac death, subsequent hospitalization for cardiac events, and emergency department visits) at 1 year in normal weight, overweight, and obese subjects with normal SE.* Results.* Excluding patients with an abnormal and indeterminate SE and those who were lost to follow-up, a retrospective analysis of 704 patients was performed. There were 366 obese patients (BMI ≥ 30), 196 overweight patients (BMI 25–29.9), and 142 patients with normal BMI (18.5–24.9). There was no MACE in the groups at 1-year follow-up after a normal SE.* Conclusions.* In obese patients including those with multiple risk factors and symptoms concerning for cardiac ischemia, stress echocardiography is an effective and reliable noninvasive tool for identifying those with a low 1-year risk of cardiac events.

## 1. Introduction

Cardiovascular disease is the leading cause of death in the United States, accounting for about one-third of all deaths in subjects over age 35 years [1]. Stress echocardiography (SE) is used widely in the evaluation of suspected coronary artery disease (CAD) and has good diagnostic accuracy as well as ability to predict prognosis [2–4]. Different studies have demonstrated that SE is accurate in the evaluation of CAD regardless of the patient's gender, clinical symptoms, and previous history of CAD [5–9]. However, acquiring echocardiographic images with good endocardial border definition that is adequate for segmental wall motion analysis may be difficult in obese subjects. Thus, the utility of SE in this population is less well verified.

There has been a dramatic increase in obesity in the United States with the prevalence nearly doubling in the past 2 decades [10]. More than one-third (35.7%) of US adults currently fall under the category of obese with body mass index (BMI) > 30 kg/m^2^ as defined by the World Health Organization [11]. Severe obesity (BMI > 35 kg/m^2^) is the fastest growing category of obesity. Obesity is associated with premature and accelerated atherosclerosis [12]. It is, therefore, important to identify appropriate diagnostic tests that help to effectively evaluate coronary risk in the growing population of obese patients.

The diagnostic utility of all cardiac imaging techniques may be limited in patients with obesity. For example, previous studies have shown that the accuracy of nuclear myocardial perfusion imaging for diagnosing CAD decreases in obese patients with a BMI > 30 [13]. Echocardiography in obese patients can result in nondiagnostic images in up to 30% of patients and clinicians may avoid using stress echocardiography in this patient population because of concerns that the test will be inconclusive [14]. However, some studies have suggested that increased BMI does not necessarily exert an adverse effect on the interpretability of echocardiographic images [15, 16].

In patients with chest pain, a normal SE was reported to have a very high negative predictive value for subsequent cardiovascular events [3]. In that study, the researchers did not include data on the BMI of the patients that were studied. There are no published data that specifically address the prognostic value of negative SE in obese patients. Given the need to confirm the utility of cardiac testing in patients with a wide range of body sizes, we have assessed one-year major adverse cardiac event rates (MACE) defined as myocardial infarction, cardiac intervention, cardiac death, subsequent hospitalization for cardiac events, and emergency department visits at 1 year after negative SE in obese and nonobese patients. We hypothesized that a normal SE would have good prognostic value in obese patients.

## 2. Methods

The study was approved by the Human Assurance Committee at the Medical College of Georgia, Georgia Regents University. We conducted a chart review of 1446 consecutive patients undergoing clinically indicated SE (dobutamine or exercise) performed from 2006 to 2009.

We used the standard 17-segment scoring model to report our findings. We defined normal as the absence of any new or worsening wall motion abnormality with stress and abnormal as either ischemia, infarction, or viable. The study was considered indeterminate if target heart rate was not reached. Inclusion criteria were a clinically indicated normal SE (defined as the absence of any new or worsening wall motion abnormality with stress and achieving ≥85% of age predicted maximum heart rate (220-age in years)) and BMI of >18.5. As per protocol, the beta blockers and calcium channel blockers were held 24–48 hours prior to elective stress testing. This was not possible when patients underwent SE as a part of inpatient or emergency department visit. Both exercise and dobutamine (with or without the use of atropine) stress protocols were used to achieve target heart rate. We collected demographic and anthropometric information, history of diabetes mellitus (DM), hypertension, dyslipidemia, smoking, peripheral vascular disease, prior stroke, CAD, previous myocardial infarction, coronary artery bypass graft, and cardiac catheterization with or without percutaneous intervention (Table 1). Overweight was defined according to World Health Organization criteria as BMI 25–29.9 kg/m^2^, obese as BMI ≥ 30 kg/m^2^, and nonobese as BMI 18.5–24.9 kg/m^2^ [17].

The aim of our study was to evaluate whether a negative SE could reliably be used to identify obese patients with low cardiovascular risk, as there are limited tools available in the evaluation for ischemic heart disease in this patient population. With this in mind, we excluded 115 patients with an abnormal or indeterminate SE, 84 and 31, respectively.

Medical records were reviewed and telephone contact was made to determine MACE within 1 year of the stress echocardiogram. Secondary outcomes included all-cause mortality, recurrent angina, emergency department visits for angina, and repeat SE. Baseline characteristics and MACE were compared between the two groups using chi square (*χ* ^2^) and Fisher's exact test. A *P* value of <0.05 was considered statistically significant. Analysis was performed using SPSS (14.0 Chicago IL).

## 3. Results

**Figure 1:**
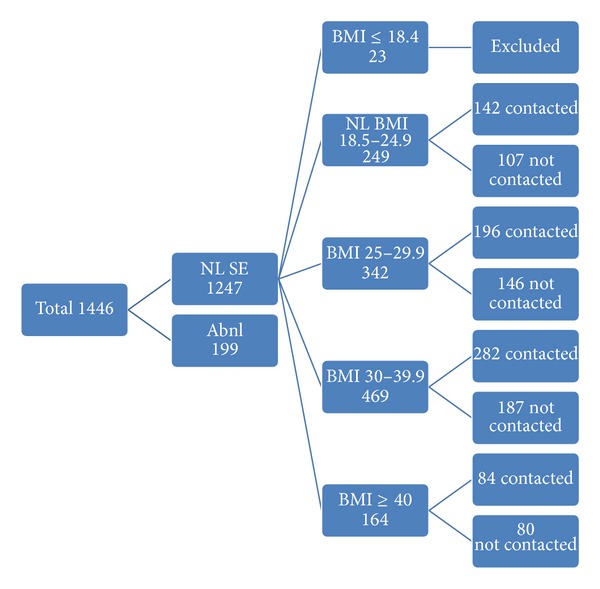
BMI = body mass index, kg/m^2^; NL = normal; Abnl = abnormal. Data are expressed as a number.

**Figure 2:**
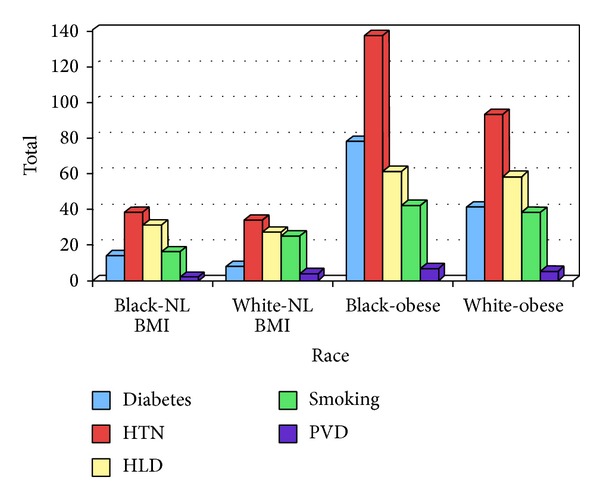
HTN = hypertension; HLD = hyperlipidemia; *n* = number; NL = normal; PVD = peripheral vascular disease, OWO = overweight and obese, and BMI = body mass index. Data are expressed as a number.

Seven hundred and four patients with 1 year of follow-up met criteria for the study (366 obese patients, BMI 37.3 ± 0.3; 196 overweight patients, BMI 27.5 ± 0.1; and 142 nonobese patients, BMI 22.6 ± 0.1; Figure 1). There were 84 patients with grade IIII obesity (BMI > 40) and 282 patients with grades I and II obesity (BMI 30–39.9). Ultrasound contrast agents were used more in the severely obese patients (grade III) compared to those with grades I and II obesity (*P* = 0.0001). More patients underwent dobutamine stress echo in the severely obese group compared to those with grades I and II obesity. Age in the normal weight patients was 53.2 ± 1.2, 53.2 ± 0.5 in the overweight group, and 50.3 ± 0.6 years in the obese group. There were more whites in the normal weight group and more blacks in the obese group (*P* = 0.01). There were significantly more patients with hypertension and diabetes in the obese versus the normal weight group (*P* = 0.019 and 0.001, resp.; Table 1). However, there was no difference in the prevalence of hypertension and diabetes in terms of race in the obese group alone (Figure 2). There was a significantly higher rate of dyslipidemia in the obese whites as compared to the obese blacks (*P* = 0.012). There were significantly more patients with hyperlipidemia in the overweight group compared to the normal BMI group (*P* = 0.01). There was a trend toward more patients with hypertension, diabetes, PVD, and CVA, although not statistically significant. There were overall more females than males in both groups and more whites in both groups. Less than 1/3 of patients in both groups were taking aspirin, beta blockers, or angiotensin converting enzyme inhibitors.

The presence of cardiovascular risk factors (hypertension, hyperlipidemia, DM, smoking, peripheral vascular disease, and prior CAD) was common. In the obese group, 18% had no risk factors, 28% had 1 risk factor, 27% had 2 risk factors, and 17% had 3 risk factors. Similarly, in the overweight group there were 14% with none, 34% with 1, 29% with 2, and 14% with 3 risk factors.

Equal numbers of normal weight, overweight, and obese patients underwent either DSE or exercise SE. However, severely obese patients were more likely to have DSE than exercise SE. All patients included in the analysis achieved at least 85% of the maximum age predicted heart rate, indicating adequate level of stress in all of the groups.

As previously mentioned, both exercise and dobutamine stress protocols were used to achieve >85% of age predicted maximum heart rate. Average dose of dobutamine was 32 *μ*g/kg/min, with 31% of patients receiving atropine. Average exercise time was 9 minutes 49 seconds, achieving an average of 9.46 METS. Approximately 4% the SE were considered indeterminate. This is likely due to inability to achieve target heart rate.

There was no MACE observed in any of the subjects who had a normal SE at 1-year follow-up, irrespective of group. There was a slight, but nonsignificant, trend towards more emergency department visits and repeat SE during the 1-year follow-up in the obese group (*P* = 0.34 and 0.735 resp.; Table 1).

## 4. Discussion

The results of this study show that normal SE has the ability to identify a low risk group of obese patients with chest pain despite having multiple coronary risk factors. The findings hold for both sexes and in both black and white subjects. Given the increasing prevalence of obesity, the risks of CAD in obese patients, and the frequent symptoms that could be ischemic in etiology, these results have important implications for day-to-day clinical decision making in real world clinical practices.

In addition to the traditional CAD risk factors of hypertension, dyslipidemia, tobacco use, and DM, prior data suggest that obesity is independently associated with both premature and accelerated atherosclerosis [12]. Obese patients commonly have symptoms of chest pain and dyspnea that are suggestive of cardiac ischemia but could also be due to direct or indirect effects of obesity. Distinguishing between these possibilities is a major but very important clinical challenge. Due to the potential limitations of cardiac imaging in patients with large body habitus, diagnostic testing to accurately delineate the etiology of symptoms and coronary risk needs to be performed with a rational approach.

The sensitivity and specificity of SE for diagnosing CAD are well established [18]. The purpose of this study was not to evaluate the sensitivity and specificity of SE in diagnosing CAD in obese patients. Rather we used clinical end points to assess whether a negative SE could adequately identify obese patients at low risk of clinical events.

Multiple noninvasive imaging modalities have been used in the diagnosis of CAD. Nuclear myocardial perfusion imaging has been well validated in predicting outcomes and guiding revascularization decisions [19]. However, in the setting of obesity, myocardial perfusion imaging is often limited by photon attenuation and scatter which can lead to poor signal to noise and attenuation artifacts [13]. Cardiac MRI and CT have shown incremental prognostic value in the setting of suspected and/or proven CAD [20, 21]. However, both of these modalities also have limitations in the setting of obesity. In the case of CT, X-ray scatter and attenuation have major effects on image quality in obese patients. Coronary CT is usually precluded in patients with BMI > 40 kg/m^2^. For MRI, bore size and/or claustrophobia are often the limiting factor in obese subjects. However, obesity may also reduce MRI image quality due to intolerance of repeated breath holding and the larger distance between the heart and the chest coils. Echocardiography also has potential problems in obese subjects, but there is no theoretical upper weight limit and image quality can be improved in many cases by the use of newer imaging platforms combined with ultrasound contrast agents [14]. In general, noninvasive testing for CAD in obese patients has been associated with lower sensitivity and specificity when compared with invasive angiography [22].

The lack of events in our cohort after 1 year of follow-up was very low. It might be argued that this finding occurred because our patients had an unusually low pretest probability of obstructive CAD. However, 82% of the obese patients had at least 1 cardiac risk factor and 44% had 2 or more. The reasons for SE were typical clinical indications that were consistent with published appropriate use criteria [23]. According to such criteria, high risk patients will generally not undergo noninvasive testing but rather proceed directly to invasive testing. A negative nuclear stress test has been reported to be associated with ~1.5% annual cardiac event rate [24]. Although our patients appeared to have a lower event rate than this, we cannot make any direct comparisons across studies using different patient populations and different imaging modalities.

Of those patients undergoing echocardiographic evaluation, the American Society of Echocardiography has suggested that ~20% of all patients have suboptimal left ventricular endocardial definition on echocardiography. Contrast enhanced stress echocardiography has been shown to increase the diagnostic value in the detection of coronary artery disease in severely obese patients [25, 26]. However, it should be noted that the use of ultrasound contrast agents has not been specifically approved by the FDA for use in stress echocardiography. In our retrospective study, ultrasound contrast agent was used in 10% in obese versus 2.8% in normal patients. The percent of contrast use in our cohort was lower than the current usage [14]. This is likely due to the fact that at the time of the data collection the use of contrast was scrupulous due to the recent FDA black box warning which led to marked declines in contrast use across the country.

Several studies have examined the influence of race and sex in the evaluation of chest pain. One study directly compared long term MACE between blacks and whites in the setting of a normal SE. Blacks were more likely to experience higher rates of nonfatal myocardial infarction and MACE compared to whites despite a normal SE [27]. Another study showed favorable outcomes in females in terms of nonfatal myocardial infarction and MACE after a normal SE [6, 7]. In these studies, the mean age was higher, duration of follow-up was longer, and African American race did confer a greater risk of MACE. In our study, we did not find any difference in terms of MACE based on gender or race. The differences between our findings and those of other published studies on this topic can likely be explained by the fact that our study population was younger and had a shorter duration of follow-up. In our study, we found that there were more whites in the normal weight group and more blacks in the obese group (Table 1). These findings are consistent with the current literature showing a higher prevalence of obesity in blacks. In our study as in others, blacks had more coronary risk factors including DM, hypertension, and dyslipidemia compared to whites (Figure 2) [28].

## 5. Limitations

The main limitation of our study is the retrospective nature with outcome data largely collected via telephone. 42% of patients could not be contacted at 1 year. However since this was a single-center study, many of the patients undergoing SE were followed longitudinally at our institution and we were able to obtain evidence of MACE (or lack thereof) from chart review. Since equal numbers of patients in each weight group were lost to follow-up, we believe our main results are valid. Demographics (age, sex, and race) were similar for normal weight, overweight, and obese groups. However, obese patients had significantly more HTN and DM and were more often of black race. Thus, it cannot be argued that the obese subjects had a lower pretest risk. It is possible that 1 year is a short time period for follow-up and this accounted for the low event rate. However, our findings are similar to those from the SPEED trial [3]. While longer duration of follow-up would have been desirable, it is almost certain that more patients would have been lost to follow-up by extending the outcome evaluation period. Our study provides roughly similar duration of follow-up to that reported previously for nuclear stress testing [29, 30]. In addition, the “warranty period” of a negative nuclear stress test has been reported to be 1 year in diabetics and 2 years in nondiabetics [31]. Thus, our study answers the clinically relevant question of whether a negative SE is a reliable short term prognostic indicator in obese patients with low to intermediate risk of coronary artery disease.

We used final clinical interpretation from the echo attending who interpreted the study and we did not do any formal testing of agreement or disagreement amongst the readers, nor did we specifically assess for any interobserver variability.

We did not specifically address side effects of dobutamine observed during the testing period. Common side effects that are felt to be pharmacological rather than ischemic in etiology (e.g., anxiety, tremulousness, and nausea) are not routinely part of our reports. The most common observations during testing were chest tightness, dyspnea, and fatigue in all groups.

In conclusion, normal SE has a good prognostic value at one year, even in obese patients with multiple cardiac risk factors. Sex or race did not affect the outcome. When image quality is considered adequate, SE is an effective and reliable noninvasive tool in the evaluation of obese patients with concern for cardiac ischemia. Importantly, a negative SE implies a low yield of additional testing in patients with recurrent symptoms, despite severe obesity.

## Figures and Tables

**Table 1:** Patient characteristics in the study population.

	Obese (*n* = 366)	Normal BMI (*n* = 142)	*P* value
Age (mean)	50.3 ± 0.6	53.3 ± 1.2	NS
Race	Black (60%)	Black (48%)	0.01
Female	69%	61%	0.09
Hypertension	64%	53%	0.019
Smoking	23%	30%	0.13
Diabetes	33%	16%	0.0001
PVD	4%	4%	0.803
HLD	29%	27%	0.585
CVA	3%	5%	0.321
Family history CAD	32%	27%	0.391
Ultrasound contrast agent used	10%	2%	0.005
Previous CAD	8%	11%	0.218
Noncardiac death	1 (0.2%)	2 (1%)	0.19
MACE	None	None	
Repeat stress at 1 year	2%	1%	0.735
ER visit at 1 year	12%	8%	0.34

CAD = coronary artery disease; CVA = cerebral vascular accident; ER = emergency room; HLD = hyperlipidemia; MACE = major adverse cardiac event; PVD = peripheral vascular disease. Data are expressed as number (percentage).

## Abbreviations

BMI: Body mass Index
CAD: Coronary artery disease
DM: Diabetes mellitus
MACE: Major adverse cardiac events
SE: Stress echocardiography.
